# Dissection of Developmental Programs and Regulatory Modules Directing Endosperm Transfer Cell and Aleurone Identity in the Syncytial Endosperm of Barley

**DOI:** 10.3390/plants12081594

**Published:** 2023-04-10

**Authors:** Christian Hertig, Twan Rutten, Michael Melzer, Jos H. M. Schippers, Johannes Thiel

**Affiliations:** 1 Department of Physiology and Cell Biology, Leibniz Institute for Plant Genetics and Crop Plant Research (IPK), D-06466 Seeland, Germany; 2 Department of Molecular Genetics, Leibniz Institute for Plant Genetics and Crop Plant Research (IPK), D-06466 Seeland, Germany

**Keywords:** barley, endosperm development, syncytium, cell differentiation/identity, endosperm transfer cells, aleurone, laser capture microdissection, RNA-sequencing, regulatory pathways, hormone signaling, transcription factors

## Abstract

Endosperm development in barley starts with the formation of a multinucleate syncytium, followed by cellularization in the ventral part of the syncytium generating endosperm transfer cells (ETCs) as first differentiating subdomain, whereas aleurone (AL) cells will originate from the periphery of the enclosing syncytium. Positional signaling in the syncytial stage determines cell identity in the cereal endosperm. Here, we performed a morphological analysis and employed laser capture microdissection (LCM)-based RNA-seq of the ETC region and the peripheral syncytium at the onset of cellularization to dissect developmental and regulatory programs directing cell specification in the early endosperm. Transcriptome data revealed domain-specific characteristics and identified two-component signaling (TCS) and hormone activities (auxin, ABA, ethylene) with associated transcription factors (TFs) as the main regulatory links for ETC specification. On the contrary, differential hormone signaling (canonical auxin, gibberellins, cytokinin) and interacting TFs control the duration of the syncytial phase and timing of cellularization of AL initials. Domain-specific expression of candidate genes was validated by in situ hybridization and putative protein–protein interactions were confirmed by split-YFP assays. This is the first transcriptome analysis dissecting syncytial subdomains of cereal seeds and provides an essential framework for initial endosperm differentiation in barley, which is likely also valuable for comparative studies with other cereal crops.

## 1. Introduction

Cereal seeds represent the major source of world’s food production. Globally, barley (*Hordeum vulgare*) is ranked second among cultivated temperate cereal crops after wheat (*Triticum aestivum* L.) [1]. Grain size and number are key determinants of crop yield in cereals and are largely determined by the growth of maternal tissues and development of the endosperm. Nevertheless, molecular mechanisms underlying cell and tissue differentiation during grain development remain poorly understood. Endosperm development in barley and other monocots follows a brief, coordinated and highly specialized pattern of cell- and tissue-specific differentiation that gives rise to the main endosperm cell types: starchy endosperm (SE), aleurone (AL), endosperm transfer cells (ETCs) and embryo-surrounding region (ESR). After fertilization of the central cell of the megagametophyte, endosperm development starts with divisions of free nuclei without cell wall formation, resulting in a multinucleate syncytium, the so-called endosperm coenocyte [2]. In barley, cellularization is initiated about three to four days after flowering (DAF) by the formation of radial microtubule systems (RMS) originating from the nuclear envelopes of free nuclei [3,4]. Phragmoplasts are inserted at the points of RMS intersection and direct cell plate assembly in the midzone to build tube-like alveolar structures. Nuclear mitotic divisions within alveoli are followed by cytokinesis to generate a first peripheral cell layer, which is succeeded by periclinal cell divisions until the endosperm is completely cellular [5]. In the ventral part of the syncytium—facing the maternal–filial boundary of grains—ETCs are formed as the first differentiating subdomain of the endosperm (3DAF), whereas the remaining, peripheral layer is still retained in the syncytial stage. The developmental gradient is maintained until repeated rounds of periclinal divisions generate peripheral cell files that will assume AL identity around 6/7 DAF. This trend of a gradient in endosperm cellularization has been reported in several dicot and monocot species, such as Arabidopsis, maize and rice [6,7], but spatial signals controlling the developmental hiatus are still largely unknown [4]. SE cells are derived from the inner daughter cells of AL, following centripetal divisions will generate newly formed cells that fill the center of the endosperm, providing the main body for starch and storage protein accumulation [4]. Contrary to other cereal grains, the barley AL consists of three layers, enriched in lipids, proteins, anthocyanins and micronutrients [8], which are also of nutritional value for feeding purposes and are agronomic traits for grain quality.

Only few molecular factors with a potential role in triggering ETC and AL cell specification and differentiation have been identified in cereal grains predominantly based on tissue-specific expression or genetic analyses. The MYB-related R1-type TF *ZmMRP-1* [9] and *ZmTCRR-1/-2* [10,11], genes of the BETL family (*ZmBETL1-4)* [12,13], *MATERNALLY EXPRESSED GENE1* (*ZmMEG1*) [14] from maize and *HvEND1* [15], *HISTIDINE KINASE1* (*HvHK1*) and type-B response regulators (*HvRRs*) from barley [16,17] are involved in ETC/maize basal endosperm transfer layer (BETL) specification. Genes associated with AL cell fate acquisition and development include *CRINKLY4 (CR4)*, *DEFECTIVE KERNEL1 (DEK1), SUPERNUMERY ALEURONE LAYER1 (SAL1), THICK ALEURONE1 (THK1),* and *NAKED ENDOSPERM1* (*NKD1*) in maize, *THICK ALEURONE2 (OsTA2)* from rice and *HvLTP2* and *ELONGATION2 (HvELO2*) from barley [18,19,20,21,22,23,24,25,26].

Several transcriptome studies characterized temporal gene expression profiles in a few subregions and/or specific tissues of seeds from various grain crops, including wheat, barley, maize and rice [27,28,29,30]. The most comprehensive study in terms of spatial resolution was generated by laser capture microdissection (LCM)-based transcriptome profiling of ten filial and maternal tissue types from maize kernels at 8 DAF [31]. Transcriptome profiles from ETCs, AL and SE cells in wheat grains have been identified at three developmental stages (10–30 DAF) [32]. Furthermore, in rice, the transcriptional profiles of SE, AL, nucellar epidermis (NE), ovular vascular trace (OVT) and cross cells (CC) were assessed at important phases of endosperm development (4, 8 and 12 DAF) [33]. However, none of these LCM-based studies addressed the pre-cellular, initial stages of the syncytial endosperm when identities of endosperm cells are set by spatial signaling [34]. A recent study dissected the first two peripheral cell layers of the rice endosperm by LCM and identified layer-specific transcriptome profiles that specify AL and SE cell fates [35]. In contrast to the concentric cellularization process in rice, cytokinesis in the endosperm of other important grain crops proceeds in dorsi-ventral directions and generates specific cell types at the maternal–filial intersection of grains (ETCs, BETLs) for transport purposes, which are reported not to exist in rice grains, making it difficult to compare details of the differentiation process.

Taken together, information about spatial molecular mechanisms determining cell fate decisions in the syncytial endosperm of cereals remains scarce. To fill this gap, we performed LCM-based RNA-seq of coenocytic subdomains of the barley endosperm just at the progression from the syncytial to cytokinetic stage (3/4 DAF). Data revealed ETCs and syncytium as distinct morphological and transcriptional domains and identified developmental and regulatory programs that coordinate the initial steps of cellular differentiation in the endosperm.

## 2. Results

### 2.1. Anatomy of the Initial Cellularization Stage of the Barley Syncytium

**Figure 1:**
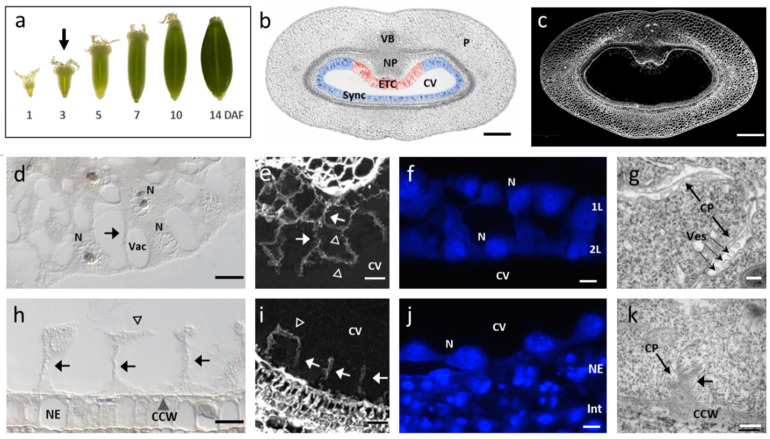
Morphological and ultrastructural analysis of the barley syncytium at the onset of cytokinesis (3/4 DAF). Light microscopy (LM, **b**,**d**,**h**), confocal laser scanning microscopy (CLSM, **c**,**e**,**f**,**i**,**j**) and transmission electron microscopy (TEM, **g**,**k**) images show details of the cellularization process in ventral (**d**–**g**) and dorsal (**h**–**k**) parts of the endosperm. (**a**) Development of barley grains until the filling stage, investigated stage marked by arrow. (**b**) Cross-section of a barley grain at 3/4 DAF, endosperm subdomains are colored: red—ETCs, blue—Sync. (**c**) Calcoflour staining of cross-sections of barley grains at 3 DAF. Magnification of LM pictures using DIC (**d**,**h**) and calcoflour-stained sections (**e**,**i**), arrows mark anticlinal, unfilled arrowheads mark periclinal cell walls, respectively. (**f**,**j**) DAPI staining of nuclei in syncytial domains. (**g**,**k**) TEM shows deposition and fusion of cell plates. CCW, central cell wall (filled arrowhead); CP, cell plate; CV, central endosperm vacuole; ETC, endosperm transfer cells; Int, integuments; 1/2L, first/second layer, NE, nucellar epidermis, N, nucleus; NP, nucellar projection; P, pericarp; Sync, syncytium; Vac, vacuole; VB, vascular bundle; Ves, vesicle. Bars = 200 µm in (**b**,**c**), 20 µm in (**d**–**f**) and (**h**–**j**), 500 nm in (**g**,**h**).

**Figure 2:**
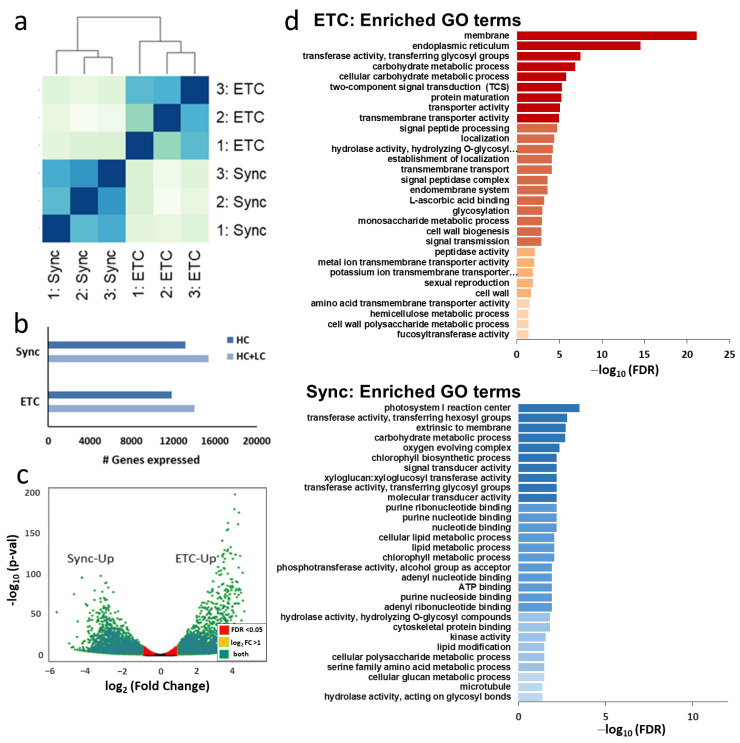
Representation of global transcriptome data from ETCs and Sync at 3/4 DAF. (**a**) Cluster dendrogram of three biological replicates from ETCs and Sync. (**b**) Number of genes expressed in ETCs and Sync. Significant expression was defined as average TPM-value > 1 in replicates. Counts for high confidence (HC) and low confidence (LC) gene models are displayed. (**c**) Volcano plot depicts significantly up- and downregulated genes between ETC and Sync according to FDR < 0.05 and log_2_ fold change > 1. (**d**) Main GO terms enriched in upregulated genes in ETCs (red) and Sync (blue). FDR values (<0.05) are −log_10_-transformed. Table S3 contains the list of all enriched GO terms (FDR < 0.05).

Around 3 to 4 DAF, cellularization of the barley endosperm was initiated. The endosperm is composed of the coenocyte, a thin layer of cytoplasm containing free nuclei, surrounding a large central vacuole (CV, Figure 1a,b). The syncytium can be morphologically separated into two subdomains: (1) the ventral part positioned at the maternal–filial boundary of grains which will differentiate into ETCs (red-labelled in Figure 1b) and (2) the remaining peripheral layer—enclosed by the nucellar epidermis (NE)—along the perimeter of the CV, which will differentiate into aleurone (AL) and starchy endosperm (SE) cells (blue-labelled Figure 1b). To characterize the morphological characteristics of subdomains, we performed light (LM), confocal (CLSM) and electron transmission microscopy (TEM). In the ETC region, the cellularization process is just initiated and has progressed to two cell layers (1L + 2L, Figure 1d). Calcoflour-white-staining of the cross-sections showed weak fluorescence signals in the syncytium compared to the maternal grain tissues, depicting cellulose accumulation predominantly in anticlinal cell walls (Figure 1c). The peripheral layer (1L) of the ETC region is bordered by anticlinal and periclinal walls, whereas the inner layer (2L, oriented towards CV) is not completely walled. In some cases, periclinal cell walls have not been formed in 2L and/or are not completely fused to anticlinal walls (Figure 1e). Fluorescence signals, particularly in 2L, are somehow patchy and fragmentary implying that the primary wall formation by cellulose deposition is still in progress. Two layers of endosperm nuclei are present and nuclei in 2L seem to be oriented towards the CV where periclinal cell walls will be formed at the cortical division zone (Figure 1f). Following rounds of anticlinal and periclinal cell divisions give rise to the three cell files (3L-structure) of differentiated ETCs, which is accomplished around 5 DAF. Ultrastructural analysis showed a dense cytoplasm which is interspersed by segments of cell plates, that are fused by vesicles at its ends to complete the cell wall formation (Figure 1g). The remaining syncytium (side and dorsal orientation) consists of a peripheral layer with multiple nuclei just before and/or during initial cytokinesis. First the anticlinal cell walls are visible, separating the cytoplasm of neighboring nuclei and providing an open-ended tube-like structure, the so-called alveoli (Figure 1h). The cellulose depositions are visible in the anticlinal walls and in rare cases, also in periclinal orientation, revealing that the formation of primary walls, i.e., cellularization of the 1L-layer, is starting around 3/4 DAF (Figure 1i). The nuclei are evenly spaced along the circumference of the syncytium adjacent to parietal cells of the nucellar epidermis (Figure 1j). As the peripheral layer is not cellularized at 3/4 DAF, it is in the following referred to as ‘Sync’. TEM depicted that cell plates are fused to the central cell wall (CCW) and build stubs that emerged in the direction towards the CV (Figure 1k). After the first round of alveoli formation, the nuclei exit the mitotic arrest and the cell plate fragments will be fused to form periclinal walls. The first walled cells of the 1L-layer will become AL. After further rounds of anticlinal and periclinal divisions, the barley endosperm is cellularized and contains three AL layers and SE cells that originated from the inner daughter cells of the peripheral AL. Altogether, the microscopy studies revealed distinct structural differences between the syncytial subdomains during the initial stages of cellularization. The ETC region displays a more advanced differentiation status, with two cell layers in the cytokinetic state, compared to the single peripheral Sync layer in the alveolar stage.

### 2.2. RNA-Seq Analysis of Genes Expressed in Endosperm Subdomains Fated to Become ETCs or AL Cells

LCM was used to isolate the ETC region and the remaining Sync from grains at 3/4 DAF (Figure 1b) for comparative RNA-seq analysis. Genome-wide RNA-seq data (with three replicates each) showed distinct clustering of the two endosperm subdomains (Figure 2a). In total, more than 14,000 genes were significantly expressed in the domains (ETC—14,000; Sync—15,400 high (HC) and low-confidence genes (LC)) according to transcript per million (TPM)-values >1 (Figure 2b). Numerous genes were differentially expressed (5703 DEGs; log_2_ fold change (FC) > 1, false discovery rate (FDR) < 0.05), with 2926 genes preferentially expressed in ETCs and 2777 genes upregulated in Sync (Figure 2c, Table S2).

Gene ontology (GO) term enrichment in DEGs depicted distinct categories transcriptionally activated in each subdomain (Figure 2d). Carbohydrate metabolism, transmembrane transport, signal transmission, signal peptide processing, cell wall biogenesis and modification, including fucose and hemicellulose metabolism, and the membrane, endoplasmatic reticulum and cell wall as cellular compartments were highly enriched in the transcriptome of ETCs. These categories reflect intrinsic features and functions of ETCs: fully developed ETCs are specialized cells with prominent walls and extensive wall ingrowths to increase the surface area for transport activities [36], which are found in seeds of a number of major crop species, including cereals and grain legumes [37]. In the signaling category, TCS phosphorelays stand out as the most enriched pathways, which is in line with the results confirming a major role in barley ETC specification and differentiation [16,17]. In contrast, lipid and chlorophyll metabolism, (ribo)nucleotide purine binding, cytoskeletal protein binding, microtubuli but also transferase/hydrolase activity (O-glycosyl compounds, xyloglucan/-glycosyls) were dominating categories in Sync. GO terms associated with photosynthesis may indicate that few cells from the surrounding (green) pericarp have also been captured by our approach. Genes involved in DNA/RNA processing, cytoskeleton organization and metabolism of xyloglucans indicate that cytokinesis is not completed, which is in line with the developmental gradient observed by histological studies. The analysis focused on DEGs assigned as HC genes to avoid confounding effects from pseudogenes and low-quality gene models (Ʃ4,521 DEGs).

#### 2.2.1. Cell Cycle Regulation, Cytokinesis and Cell Wall Formation Differ between ETC and Sync

**Figure 3:**
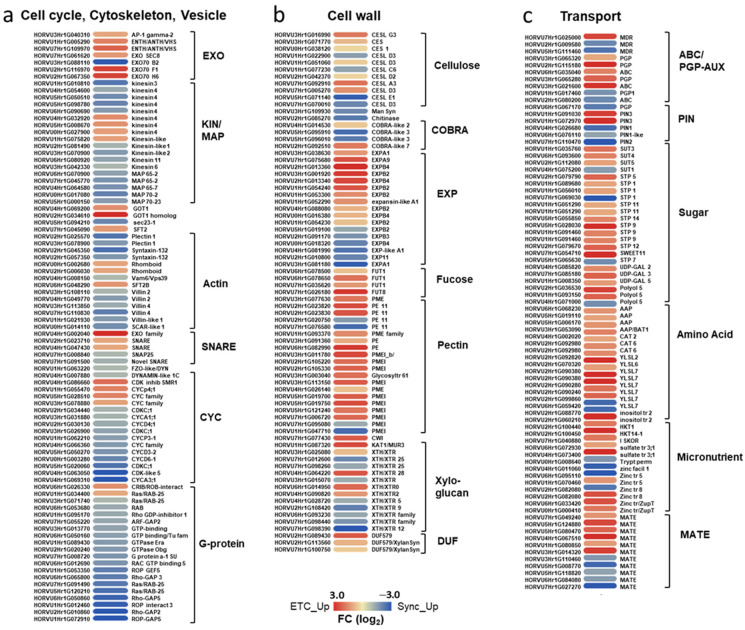
Heatmap of DEGs between ETCs and Sync at 3/4 DAF related to cell cycle regulation, cytoskeleton formation, vesicle transport, cell wall metabolism and transport. (**a**) Genes involved in cell cycle regulation, cytoskeleton formation and vesicle transport. (**b**) Genes involved in cell wall metabolism. (**c**) Genes involved in transmembrane transport. DEGs were identified according to FDR < 0.05 and log_2_ fold-change > 1, log_2_-ratios are depicted by color code: red—upregulated in ETCs, blue—upregulated in Sync. Table S4 contains the list of DEGs in the different categories with fold-changes and FDR.

Mitosis, cytokinesis and cell wall formation are interrelated processes determining cell number, cell size and final endosperm shape. Multiple genes involved in cell cycle control, cytoskeleton formation and vesicle trafficking pathways are differentially expressed between ETCs and Sync. Coinciding with the GO term enrichment, the majority of genes is predominantly expressed in the Sync layer. Seven cyclins from the A- and D-type (*HvCYCA, HvCYCD*) and four genes encoding cyclin-dependent kinases (*HvCDKs*) are upregulated in the Sync emblematic for an activated cell cycle. Cyclins from different subgroups build complexes with CDKs to control the cell cycle progression [38]. Several microtubule-associated proteins (MAPs), microtubule-binding motor proteins (kinesins), actin-binding plectins and villins underline activated cytoskeleton and spindle microtubule organization/rearrangement in Sync (Figure 3a). Villin proteins bind and bundle F-actins in a calcium-dependent manner, plectins stabilize actin filaments and act as regulators of cellular processes involving actin filament dynamics. Targeted vesicle trafficking throughout the endomembrane system is essential for cytokinesis and the establishment of cell polarity. Vesicle formation is regulated by G-protein signaling with small GTPases as molecular switches between GTP/GDP-bound states that are controlled by ARF guanine-nucleotide exchange factors (ARF-GEFs) and GTPase-activating proteins (GAPs) [39]. The preferential expression of ARF-GEFs, numerous Ras-related-/RAB-GTPases, GTP-binding and GAPs in Sync reveals activated membrane trafficking required for cell plate adhesion/insertion during the formation of initial syncytial walls. In ETCs, four genes encoding components of the exocyst complex (*HvEXO70F1/-H6, HvSEC8*), four *HvGOT1/SFt2*-like vesicle transport proteins, Rhomboid transmembrane proteins and two *HvSNARE* proteins are upregulated. Exocyst subunits are involved in the fusion of the post-Golgi secretory vesicles with plasma membranes and play a major role in in exocytosis. Golgi localized GOT1/SFT2-like membrane proteins are thought to interact with SNARE proteins that govern the fusion to vesicle target membranes for the addition of material to membranes and cell plates. Such a fusion process is shown in Figure 1g. Transcriptional activities depict that nuclei in the Sync layer are in late mitotic stages and show signatures of initial cytokinesis as deduced from cytoskeleton activities and vesicle trafficking. This corresponds to the transient alveolar stage of Sync in contrast to nearly walled ETCs.

Genes encoding enzymes implicated in cell wall formation and biogenesis are preferentially expressed in ETCs (Figure 3b). Particularly, multiple genes related to cell wall modification and cell elongations are upregulated in the ETC region. Sixteen genes associated with pectin synthesis, such as pectin(methyl)esterases (PE/PME), pectin lyases (PLs) and pectin metabolism (pectin methylesterase inhibitors/PMEI) suggest a pivotal role for the pectin matrix in ETC formation. Despite being found in low concentrations in cereal endosperm cells [40], pectins represent—together with cellulose and hemicellulose—the third main group of wall polysaccharides, as they provide the matrix in which hemicelluloses and cellulose fibrils are embedded [41]. The secretion of pectins was reported to be essential for cell plate biogenesis and transformation into the cell wall [42]. Remarkable is the coincidence of the enriched GO term ‘fucosyltransferase act.’ (GO:0008417) and the strong expression of four genes encoding fucosyltransferases (*HvFUTs*) in ETCs, which are specific glycosyltransferases involved in the fucosylation of xyloglucan. L-fucose is a key component in the association between cellulose and xyloglucan by enhancing the binding-affinity for cellulose microfibrils [43] and FUTs control cell adhesion during cell division [44]. A further gene strongly upregulated in ETCs (HORVU1Hr1G038500) encodes the xyloglucan galactosyltransferase (XGT) KATAMARI1/MUR3 homolog, *mur*-mutants exhibit distorted microfibril deposition and cell elongation [45]. Several cellulose synthases (*HvCES1*) and CES-like (*HvCESL A/D/G*) homologs are preferentially expressed in ETCs, despite other *CESL* isoforms are also upregulated in Sync. Most of the CESL proteins are required for the synthesis of non-cellulosic wall polysaccharides. Subsequently, hemicellulose rather than cellulose synthesis could be initiated in the Sync layer, which is in line with weak cellulose signals in stained sections (Figure 1i). The preferential expression of xyloglucan endotransglycosylases in Sync indicates hemicellulose modification and turnover. Differences between both endosperm domains are also seen in cell elongation, which is constituted by wall-loosening expansins. In particular, alpha- and beta-expansin (EXP) isoforms are preferentially expressed in ETCs). In summary, cell wall formation and further modification, including the crosslinking of cellulose, hemicelluloses, pectins and cell expansion is activated in the ETC region.

#### 2.2.2. Assimilate Transport Is a Key Feature of ETCs

The main function of ETCs is to ensure nutrient transfer from maternal tissues into the endosperm via an apoplasmic barrier [36]. In particular, active nutrient uptake is required for the provision of hexose sugars and amino acids to fuel cell proliferation and growth in the initial phases of endosperm development. Associated with transmembrane transport, a wide array of sugar, amino acid and potassium transporters are significantly upregulated in the ETC region (Figure 3c). Sixteen monosaccharide transporters, among them eleven H+/hexose cotransporters (*HvSTPs*), two polyol/monosaccharide transporters and three UDP-galactose transporters, were highly enriched in ETCs. *HvSTP1* has been shown to be expressed in very early endosperm development (from the syncytial stage onwards) and to be spatially and temporally associated with the cell wall invertase1 (*HvCWIN1*), suggesting an interplay between the liberation of hexoses by invertase activity and active uptake by HvSTP1 [46]. Three sucrose transporters—(*HvSUT3/-4/-5*) and *HvSWEET11* (HORVU7Hr1G054710)—are preferentially expressed in ETCs, whereas *HvSUT1* (HORVU4Hr1G075200) showed a higher transcript abundance in Sync. HvSUT1 is a well-known key transporter for sugar transport into the endosperm with important implications for grain filling [47]. Moreover, a suite of amino acid permeases (*HvAAPs*), cationic amino acid transporter (*HvCAT2/-6*) and *YELLOW STRIPE*-like transporter (*HvYSLs*) are upregulated in ETCs. YSL proteins transport nicotianamine and zinc/iron ions which are essential nutrients limiting plant growth. Other groups of transporter genes preferentially expressed in ETCs comprise inositol and sodium (*HvHKTs*) transporters that are also controlling the activity of sucrose transporters by regulating the osmotic potential [48]. In contrast, a sulfate transporter, two multidrug resistance-associated (MRP) family proteins and six MATE efflux proteins show predominant expression in Sync. Several isoforms of auxin efflux carrier proteins (*HvPIN* genes) are conversely expressed in both tissues. Two barley orthologs of Arabidopsis PIN3 are upregulated in the differentiating ETCs, among them is HORVU1Hr1G072970 with a fold change of >18, whereas three isoforms of PIN1 and PIN2 are preferentially expressed in Sync. Reciprocal expression is also seen for family members of the phosphoglycoprotein (PGP/ABC) auxin transporters (4 isoforms in ETCs vs. 3 in Sync), which are interacting with PIN proteins in asymmetric auxin distribution [49]. Together, the expression patterns of transport-related genes verified the role of the ETC region as a bottleneck for nutrient transfer from maternal tissues into the filial part of the grain. The active transport of bulk-flow assimilates, i.e., hexoses, sucrose and amino acids, but also micronutrients, facilitated by membrane-bound transporter proteins seems to be already established when cells acquire ETC identity, before they are fully differentiated.

#### 2.2.3. Signaling Pathways Directing Cell Specification in Endosperm Domains

##### Different Hormone and Phosphorylation Pathways Are Transcriptionally Activated in Syncytial Subdomains

**Figure 4:**
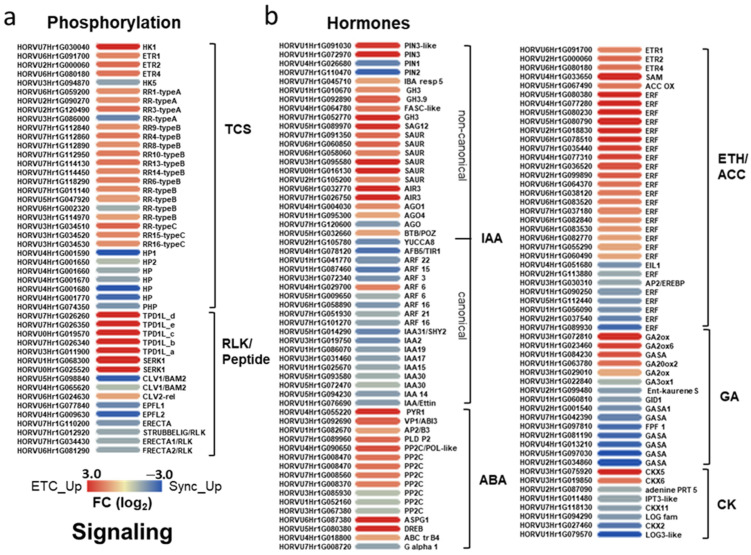
Heatmap of DEGs related to signaling pathways in ETCs and Sync at 3/4 DAF. (**a**) Genes involved in phosphorylation and peptide signaling. (**b**) Genes involved in hormone signaling. DEGs were identified according to FDR < 0.05 and log_2_ fold-change > 1. The log_2_-ratios are depicted by color code: red—upregulated in ETCs, blue—upregulated in Sync.

Among the signaling pathways, TCS phosphorelays stand out as the most enriched category (FDR < 4.9 × 10^−6^) in differentiating ETCs (Figure 2d). Four histidine kinases (HKs), including three putative ethylene receptors *HvETR1/-2/-4* and *HvHK1* as prominent example for a cytokinin-independent HK with a key function in ETC specification [17], were highly expressed in ETCs (Figure S1). Response regulators (RRs) represent the output components of TCS and are deemed to confer hormonal signals in a cell/tissue-specific manner. Three type-A RRs, ten type-B RRs and three type-C RR genes were preferentially expressed in ETCs (Figure 4a), revealing an interaction between different subgroups of RRs during the initial cellularization steps of ETCs. In contrast, only *HvHK5* (HORVU3Hr1G094870), a putative cytokinin (CK) receptor with a CHASE domain, one type-A and type-B RR were upregulated in Sync. Unexpectedly, several His-containing phosophotransfer proteins (HPs) were preferentially enriched in the Sync layer. HPs are characterized as intermediate elements in phosphorelays that interact redundantly with different HKs and RR subgroups and thus, participate in multiple TCS modules [50]. Nevertheless, TCS signaling in Sync is attenuated compared to ETCs, and only few elements potentially involved in CK signaling are transcriptionally activated in Sync. Among the numerous leucine-rich receptor-like protein kinases (LRR-RLKs) expressed in both domains, two barley orthologs of *CLAVATA1*-related *(HvCLV1/BAM2)* receptor kinases (HORVU5Hr1G098840, HORVU4Hr1G065620), three *ERECTA* (*HvER1/-2/ER-LIKE1*) genes and *HvSTRUBBELIG3* were preferentially expressed in Sync, together with ER/ERL peptide ligands *EPIDERMAL PATTERNING FACTOR-like1/-2* (*HvEPFL1/-2*). Contrastingly, two genes encoding *SOMATIC EMBRYOGENESIS RECEPTOR-LIKE1* (*HvSERK1*) and five *TAPETUM DETERMINANT1*/-*like* (*HvTPD1/HvTPD1L*) genes were highly expressed in ETCs.

An array of genes involved in hormone metabolism, perception and signaling (auxin, abscisic acid (ABA), ethylene, gibberellins (GA), CK) is found in the set of DEGs (Figure 4b). The highest number of DEGs are probably associated with auxin and ethylene signaling. Multiple auxin responsive genes, such as *GH3s*, *SAURs* and *IBA response 5*, are concomitantly upregulated in ETCs, accompanied by *HvTPD1L* homologs, *ARGONAUTE1+-4* (*HvAGO1/-4*) and two *AUXIN-INDUCED IN ROOT CULTURE3* (*HvAIR3*)/Sub-protease orthologs. *TPD1*-like genes encode cysteine-rich peptides (CRPs) involved in microspore and ovule development and work upstream of auxin signaling and core cell cycle genes [51]. AIR9 was detected as a microtubule-binding protein essential for correct cell plate orientation in Arabidopsis [52], *AtAGO1* encodes a repressor of *ARF7* and *ago1*-mutants show pleiotropic morphological defects. Together with HvPIN3 efflux carriers, these elements might contribute to a specific branch of non-canonical auxin regulation in ETCs. Three putative ethylene receptors (*HvETR1/-2/-4*), enzymes putatively involved in ethylene biosynthesis (SAM-methyltransferase, ACC oxidase (OX)) and a suite of ERF transcription factors (19 genes), mostly from the B-2 subfamily of ERF/AP2 TFs are commonly upregulated in ETCs. A stimulated expression in ETCs is also obvious for elements involved in ABA signal transduction; ABA receptor *HvPYR1/RCAR*, *PHOSPHOLIPASE αD2* (*PLD D2*), *HvABI3*/-*Rav2*-like, several members of the PP2C family, *HvDREBB1* and ABC transporter B4 support ABA influences.

In contrast, genes involved in auxin, CK and GA metabolism and signaling showed a pronounced transcriptional activity in Sync. In general, combinatorial interactions of auxin, CK and GA signaling pathways control cell division and cell differentiation processes. *HvYUCCA8* (HORVU2Hr1G105780) is the only auxin biosynthesis gene that is preferentially expressed in Sync and seems to be functionally analogous to endosperm-specific *OsYUCCA11* and *ZmYUCCA1* [53]. Moreover, all elements participating in canonical auxin signaling pathways are strictly upregulated: putative receptor *AUXIN-RELATED F-BOX5 (HvABF5*) from the TIR1 family, seven *AUXIN RESPONSE FACTORS* (*ARFs*), and nine AUX/IAA genes point to activated auxin signaling pathways in the Sync layer during the progression of the syncytial to cytokinetic phase. CK metabolism genes, such as *ISOPENTYLTRANSFERASE3* (*HvIPT3*), *LONELY GUY3* (*HvLOG3*), *LOG* family protein and cytokinin dehydrogenases (*HvCKX2/-11*) were upregulated in the syncytial layer (Figure 4b). IPT3 catalyzes the initial step of cytokinin biosynthesis to produce iP nucleotides, LOG proteins convert inactive cytokinin nucleotides to the biologically active form, whereas CKXs catalyze the degradation of CK [54]. Together with the preferential expression of *HvHK5*, high activity of cytokinin production and turnover supports regulatory associations between CK signaling and progression to cytokinesis in Sync. Although some genes attributed to GA biosynthesis (GA2/-20-oxidases (*HvGA2OX1/-6, HvGA20OX*)) are also expressed in ETCs, GA formation, perception and signaling dominates in Sync. *Ent*-kaurene synthase, a key enzyme of GA biosynthesis, Hv*GA3OX1, HvGID1*, an ortholog of the rice receptor gene *OsGID1,* and six genes encoding GA-regulated GASA proteins are highly upregulated in Sync. *FLOWER PROMOTING FACTOR1* (*FPF1*/HORVU3Hr1G097810), shown to modulate flowering time by affecting the GA signaling pathway [55], is also enriched in Sync. Together, the activities of hormone-related genes and spatial intersections illustrate the combinatorial complexity of the regulation that coordinates cell fate decisions within the developing syncytial endosperm.

##### Signatures of TF Families Differ Substantially between Syncytial Domains

**Figure 5:**
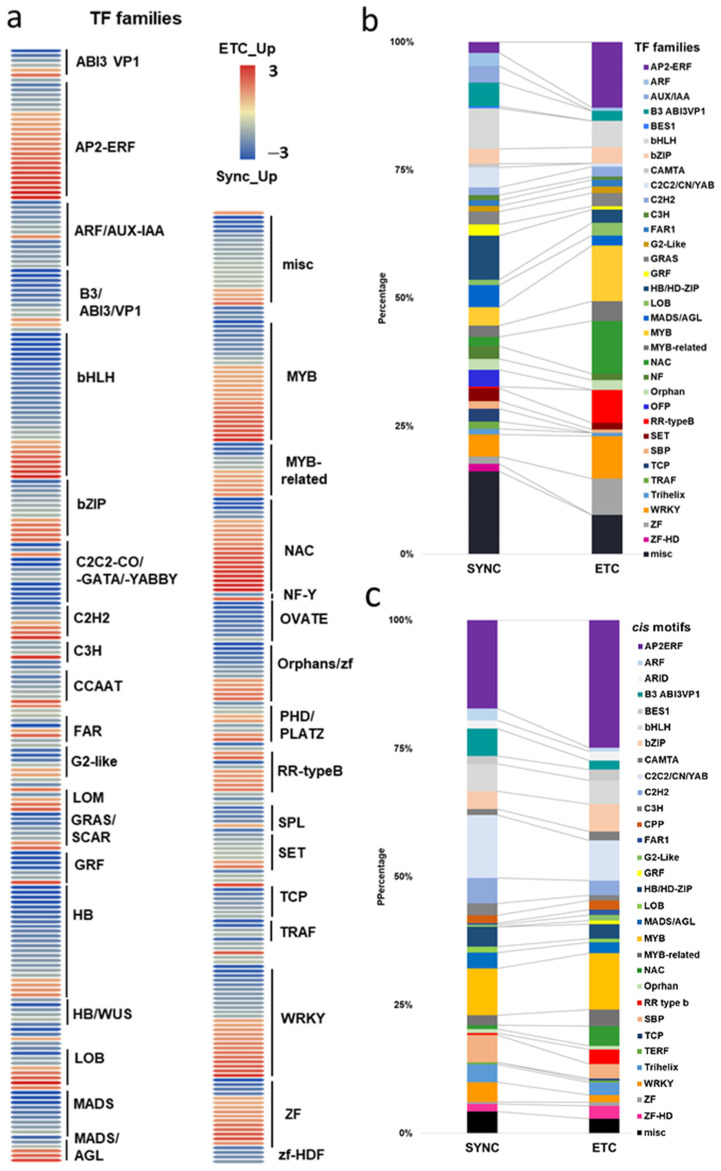
Heatmap of DEGs encoding transcription factors (TFs); representation of TF families and *cis*-regulatory motifs in ETCs and Sync at 3/4 DAF. (**a**) Heatmap for all DEGs encoding TF genes (FDR < 0.05, log_2_ FC > 1). (**b**) Distribution of TF families given as percentage from all upregulated TFs in Sync (278 genes) or ETCs (157 genes), respectively. (**c**) Abundance of *cis*-motifs in promoter regions (2000 bp upstream of ATG start codon) of upregulated genes in Sync and ETCs, only *cis*-motifs occurring in at least 5% of the sequences are displayed. Representation is given as percentage from all regulatory DNA sequence motifs.

Besides genes connected to TCS phosphorelays, peptide and hormone signaling, a significant number of transcripts (435 genes) encoding for transcription factors (TFs) are differentially expressed between both subdomains. A large proportion of the TF families showed distinct expression in either ETCs or Sync, indicating that different transcriptional networks are connected to cell identities (Figure 5a,b). In Sync, 278 genes encoding TFs are preferentially expressed, comprising 11.6% of the upregulated genes (2407), whereas the number of ETC-specific TF genes is substantially lower (157 genes/7.5% of the upregulated gens).

AP2/ERF, zinc finger/ZF, NAC, MYB TFs and also type-B RRs (working as TFs) are the few dominating TF families in ETCs with multiple members showing an enriched transcriptional activity compared to Sync (Figure 5a). In congruence with the stimulated ethylene perception and signaling, a range of *AP2/ERF* genes are distinctively upregulated in ETCs, most of them being members of the B-2 subfamilies (RAP2.2/−2.12) and drought response element binding (DREB) TFs. RAP2 proteins work as transcriptional activators through the GCC box and acted downstream of EIN2 and CTR1, whereas DREBs were also shown to be involved in ABA responses. ABI3/VP1 is a confirmed central regulator of ABA signaling in Arabidopsis embryos [56], which is also true for ABRE-binding bZIP proteins and WRKY TFs. Besides the high abundance of type-B RRs (containing a MYB domain), 23 *HvMYB/-related* TFs are enriched in the ETC transcriptome. R2R3 family proteins were shown to be involved in immune responses, but also in phase transitions from vegetative to reproductive development. Remarkably, three of the MYB TFs upregulated in the ETCs (HORVU7Hr1G024150, HORVU7Hr1G028140, HORVU7Hr1G038390) belong to the Circadian Clock Associated 1-like (CCA1-like) subgroup and are orthologs of ZmMRP-1, a well-known master regulator of BETL development in maize [9]. A recent phylogenetic analysis revealed that ZmMRP-1 orthologs are also existing in other important cereal crops, such as rice and wheat, and showed expression in young grains and/or ETCs [57]. NAC TFs are confirmed to play a role in stress responses, particularly for osmotic stress or oxygen limitation. Along interactions with stress-related hormones (ABA, ethylene), a role in developmental processes, including seed formation, was also shown for NAC proteins [58]. Sixteen *HvNAC* genes highly expressed in ETCs hint at an important function in the cellularization of ETCs in a putative crosstalk with ABA and ethylene signaling elements.

In Sync, beside ARFs-AUX/IAAs, twenty-two basic HELIX-LOOP-HELIX (bHLH) TFs, B3 domain proteins, C2C2 CONSTANS (CN)-like/C2C2-GATA, four YABBY genes, NUCLEAR TRANSCRIPTION FACTORs Y (NF-Y), GRAS family genes including SCARECROW-like proteins (SCLs), GROWTH REGULATING FACTORs (GRFs), HOMEOBOX proteins (HB), WUSCHEL (WUS)-related HBs (WOX), MADS-box, OVATE FAMILY PROTEINS (OFPs), SQUAMOSA PROMOTER BINDING proteins (SPL), SET, TRAF and TEOSINTE BRANCHED1/CYCLOIDEA PROLIFERATING CELL FACTOR (TCP) TFs are preferentially expressed. Spatial intersections of hormone activities (auxin, CK, GA) in Sync are probably connected to transcriptional programming constituted by TFs from distinct families. The upregulation of canonical auxin signaling elements in the barley syncytium, including multiple ARF TFs (*HvARF3/−5/−15/−16/−21*) and nine *AUX/IAAs*, among them *ETTIN*, the *SHY2* orthologs known to be involved in the patterning processes of reproductive organs and roots, underline enhanced auxin activity. Maize *NKD1* and *NKD2* encode duplicated INDETERMINATE DOMAIN (IDD) C2H2 TFs, named *ZmIDD9/-veg9*, and a transcriptome analysis of the double mutant *nkd1,2* uncovered ARFs as hubs in co-expression networks that define aleurone specification and differentiation [59]. Remarkably, a barley *HvNKD1 + 2* (HORVU4Hr1G070950) ortholog is strongly upregulated in Sync, thus reflecting cell specification in the peripheral layer where nuclei are fated to become AL cells. Other TFs associated with auxin signaling are the B3 domain and bHLH proteins. Several isoforms of the *MATERNAL EFFECT EMBRYO ARREST45 (HvMEE45)/B3* domain are preferentially expressed in Sync, and MEE45 controls cell proliferation and seed size by regulating auxin biosynthesis in ovule integuments of Arabidopsis. Several members of the bHLH gene family are part of a genetic network that mediates auxin signaling in suspensor development [60]. With more than 20 *HvbHLH* genes upregulated in Sync, a role in auxin signaling pathways can be anticipated.

Numerous TFs activated in Sync are documented to be involved in stem cell maintenance, meristem determination, the establishment of organ polarity or phase transitions in Arabidopsis. Upregulated isoforms of *SCL* genes from the GRAS family, *CN-like* zinc fingers, *KNOTTED1-like11 (HvKNOX11*) and *SPL* TFs are potentially involved in GA signaling pathways. SCLs are positive regulators of GA signaling by repressing the activities of DELLA proteins, CN proteins promote flowering and interact with DELLAs, KNOX1 regulates the GA pathway via the repression of the GA-biosynthesis gene *AtGA20ox1*, whereas the expression of *SPL* TFs is affected by GA and a role in reproductive phase transitions has been documented [61]. Five genes encoding GRFs (*HvGRFs*) and *GRF1 INTERACTING FACTOR3 (HvGIF3*) are specifically upregulated in Sync (*HvGRF1/−4/−5/−6* with >10-fold enrichment). GRFs were identified as GA-inducible genes [62] and are expressed in actively growing tissues [63]. Seven *TCP* family genes are specifically expressed in Sync. Other Sync-specific TFs related to cell/organ elongation and differentiation are OFPs (nine *HvOFPs* strongly upregulated) and SET domain proteins. SET domain proteins act as histone methyltransferases and belong to the Polycomb Repressive Complex 2 (PRC2), which controls flowering time via the repression of floral promoting and floral identity genes. Twelve barley *MADS-box* TFs, including three *HvAGL* genes, are highly upregulated in Sync, which are confirmed players in specifying floral organ and ovule development in Arabidopsis. Among *HvAGL6a/-6b* and *HvAGL80*, the Arabidopsis ortholog AGL80 was shown to function as a key TF required for central cell and early endosperm development [64]. Twenty-four homeobox TFs (*HvHBs)* are preferentially expressed in Sync, including four WUS-related genes (*HvWOX2/-7* isoforms). Other genes of HB subgroups comprise *HvBEL1-like/POX, HvGLABROUS* and *HvHOX/REVOLUTA-like* genes. BEL1-like proteins restrict *WUS* expression and interact with AGL, KNAT1/-3 and OFP1 proteins. *HvGLABROUS1/-7/-11* are distinctively upregulated in Sync, and orthologs in Arabidopsis have been reported to regulate epidermal cell fate determination function in roots, shoots and seeds [65]. The WUS-CLV feedback loop generally controls stem cell maintenance and restricts cell and organ differentiation [66]. A repressory function for the meristematic activity and growth inhibition of lateral organs via an interaction with KNOX and MADS-box proteins was also elucidated for YABBY TFs from which *HvYABBY2/-3/-5* isoforms, including *HvCRABS CLAW*, are also upregulated in Sync. 

In summary, transcriptional signatures of TF families differ substantially between both endosperm subdomains and might integrate distinct hormone signals. Developmental fates and the timing of cellularization seems to underly hormonal activities associated with cell cycle regulation, cytokinesis and cellular differentiation.

### 2.3. Enrichment of Cis-Regulatory Motifs in DEGs Specifies Targets of TFs 

The promoter regions (2000 bp upstream of the ATG start codon) of the top 500 ETC- or Sync-upregulated genes were analyzed by Homer [67] to identify TF binding sites (TFBS). Identified enriched motifs for both tissues are listed in Table S5. The enrichment of sequence motifs for specific TFs in each TF family are presented in Figure 5c and show specific differences between the ETC and Sync (Figure 5c). Moreover, the *cis*-element analysis largely correlated with the preferentially expressed TFs/-families (same coloring in Figure 5b,c). Motifs bound by AP2/ERF, MYB, MYB-related, NAC and type-B RR (ARRs) TFs were enriched in promoters of ETC-expressed genes and corresponded to activated TFs involved in ethylene and ABA signaling and the pronounced expression of TCS genes. 

A clear distinction was found for *cis*-motifs in Sync, ARF, B3, C2H2, C2C2, HB/HD-ZIP, MADS/AGL SPL/SBP and WRKY binding sites that were more abundant, reflecting the expression of respective TF family members in Sync-specific transcriptional networks. The top enriched motifs (Table 1) in potential targets differed also between the ETCs and Sync. ERF15 + ERF6 and MYB41/-3/101 binding sites known from Arabidopsis are highly enriched in ETCs (6- to 10-fold). ERF 15/-6 TFs are implicated in ethylene signaling and work upstream of MAPK(K) cascades. MYB 41 and 101 are reported to be required for stress responses and are positive regulators of ABA signaling. Two CCA1 and LCL1 motifs bound by MYB-related TFs were also found in the top motifs. CCA1 motifs are bound by MRP-1/-like TFs from which three family members are upregulated in the ETCs underlining a pivotal role for controlling multiple genes during ETC specification, similar to the key function of ZmMRP-1 in BETL differentiation. Further, it was shown that the overexpression of *CCA1* strongly influences spike and grain development in wheat [68]. Moreover, two Arabidopsis RR motifs (ARR1 + 10) are enriched in ETCs’ expressed genes, showing a high similarity to barley type-B RRs (*HvRR4/-8/-10*) that are upregulated in ETCs. Remarkably, CHIP-seq experiments identified genome-wide targets of ARR1 and ARR10, which are involved in ABA, ethylene and auxin signaling pathways, aside from CKs [69]. Relying on functional conservation in monocots, a similar role in interacting pathways coordinating barley ETC specification can be anticipated. In Sync, the barley HVH21 motif is the most enriched TFBS (>13-fold). Gel shift experiments showed that the TGAC element of HVH21 is required for binding by HOODED, a KNOTTED-like HD TF [70], coinciding with the pronounced expression of *HvKNOX11* (HORVU7Hr1G114650). Other enriched motifs were documented to be targets of MADS, SPL13, bZIP, C2H2, C2C2 Dof and ARF36 TFs (Table 1). MADS-binding sites include recognition sequences of the SEP AGL subgroup, namely Arabidopsis SEP3 and tomato AGL1 motifs; StAGL1 affects fruit ripening and AtSEP1/-3 are functionally redundant in directing petal/stamen identity. Several AGL isoforms, *HvSEP1/-3* (HORVU5Hr1G095710, HORVU7Hr1G012920) and *HvAGL6/-80*, are upregulated in Sync, besides *HvSPL13/-16/-18/-23* genes*,* C2H2 TFs, including *HvNKD1/2*, CN-like, GATA and YABBY C2C2 zinc finger proteins and *HvARFs*. All of these TFs have in common an involvement in cell determination and/or developmental phase transitions and imply regulatory associations with GA and auxin signaling. Together, the analysis of *cis*-regulatory motifs revealed a high commonality between the enrichment of target sequences and transcriptional profiles of TF family genes during the acquisition of cell identities. Top-enriched motifs in promoter regions of DEGs probably specify master regulators that activate multiple domain-specific target genes.

### 2.4. In Situ Hybridization Validates Domain-Specific Expression

**Figure 6:**
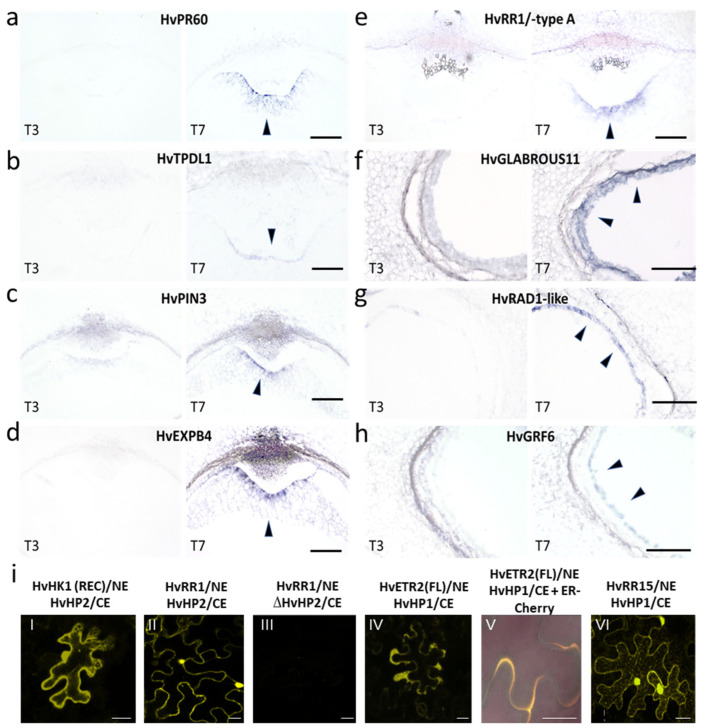
Validation of subdomain-specific expression by in situ hybridization (ISH) and complex formation of TCS elements. (**a**–**h**) ISH of ETC- and Sync-specific genes in 3 DAF barley grains. Sense (T3/negative control) and antisense (T7) probes are shown on the left and right side of panels, respectively. (**a**–**e**) ETC marker genes (HvPR60, HvTPDL1, HvPIN3, HvEXP4, HvRR1); (**f**–**h**) Sync marker genes (HvGLABROUS11, HvRADL1, HvGRF6). Arrowheads indicate main ISH signals. Bars = 100 µm. (**i**) Bimolecular fluorescence complementation (BiFC) of barley TCS elements in tobacco leaves; NE, N-terminal part of YFP with interaction partners HvHK1 (REC domain), HvRR1, HvETR2 (full length), and HvRR15; CE, C-terminal part of YFP with interaction partners HvHP2, ΔHvHP2 (mutated), and HvHP1; ER-Cherry, Cherry-marker for endoplasmatic reticulum [73]; white bars = 20 µm.

In situ hybridization was performed on cross-sections of barley grains at 3 DAF to confirm the ETC- and Sync-specific expression of selected candidate genes. *HvPR60* (HORVU1Hr1G012870), an ortholog of wheat PR60 encoding an ETC-specific cysteine-rich protein [71], highly expressed *HvTPDL1* (HORVU0Hr1G019570), auxin efflux carrier *HvPIN3* (HORVU1Hr1G072970) similar to BETL-specific *ZmPIN1b* [72], *HvEXPB4* (HORVU2Hr1G013360) and type-A RR (*HvRR1*, HORVU6Hr1G059200), with a proposed role in the initial steps of ETC cellularization [30], were exclusively expressed in the ETC region (Figure 6a–e). The signals were restricted to a few cell files of cellularizing ETCs adjacent to the maternal–filial boundary of the grains. *HvEXP4* and *HvPIN3* appeared to be more strongly expressed in the inner cell layer (1L) where cells are fully walled and periclinal cell walls have been formed. Highly enriched TFs in Sync (>10-fold) were predicted to be important for further AL specification from GRF. HB and RADIALIS families were used for expressional confirmation. Signals for *HvGRF6* (HORVU4Hr1G010080), potentially involved in GA signaling, *HvGLABROUS11* (*HvGL11*, HORVU6Hr1G079410), a HB gene belonging to the HD-ZIP IV family from which several GL genes are involved in epidermal cell fate acquisition, and *HvRAD1-like* (HORVU1Hr1G089510) belonging to a small subfamily of single-MYB TF genes, were confined to the peripheral Sync layer of the endosperm (Figure 6f–h). Only *HvRAD1-like* shows some background signals in the maternal chlorenchyma (Figure 6g). Sync-specific expression and proposed functions identified them as novel marker genes for AL fate in the barley endosperm. 

### 2.5. Bimolecular Fluorescence Complementation (BiFC) Assays Confirmed Complex Formation of Co-Expressed Barley TCS Elements 

Transcripts of several TCS elements, HKs including putative ethylene receptors (ETRs), and RRs from different subgroups, were highly enriched in ETCs and pinpointed to functional relations, i.e., interactions in specific phosphorelays with intermediate HP elements. Physical interactions were observed for the REC domain of HvHK1 (HORVU7Hr1G030040) and the putative downstream cognate histidine-containing phosphotransfer protein (HvHP2, HORVU4Hr1G001650) in the cytoplasm (Figure 6i(I)). A complex formation was also observed for HvHP2 and HvRR1 (HORVU6Hr1G059200) predominantly in the nucleus and to a minor extent in the cytoplasm (Figure 6i(II)). The specificity of the interaction was confirmed by co-infiltration of HvHK1 with a mutated ΔHvHP2 element in which the phosphoacceptor site was modified and no fluorescence signal was observed in this combination (Figure 6i(III)). Assays with the full-length ethylene receptor HvETR2 (HORVU2Hr1G000060) gave a strong signal when co-expressed with HvHP1 (HORVU4Hr1G00159) that overlapped with signals of the endoplasmatic reticulum (ER) marker [73]. The ER localization of the HvETR2-HvHP1 interaction (Figure 6i(V)) was in line with localization studies of Arabidopsis ETR/ERS receptors [74]. The complex formation of HvHP1 and HvRR15 (HORVU3Hr1G034520) was localized predominantly in the nucleus resembling the HvHP2–HvRR1 complex (Figure 6i(VI)). Results show that the TCS elements transcriptionally activated during ETC specification are able to interact in vivo in phosphorelay modules, and thereby underline the value of the transcriptome data to extract regulatory elements that participate in common signalling cascades.

## 3. Discussion

Positional signaling in the syncytial stage directs cell specification and cell identity in the early endosperm. ETCs and AL cells originate from the same primary triploid endosperm nucleus, but information about the spatial molecular mechanisms determining cell fate decisions in cereal endosperms remains scarce. To fill this gap, we performed LCM-based RNA-seq of coenocytic subdomains of the barley endosperm just at the progression from the syncytial to cytokinetic stage (3/4 DAF). Data revealed the ETCs and Sync as distinct morphological and transcriptional domains and could identify developmental and regulatory programs that coordinate the initial steps of cellular differentiation in the endosperm. The analysis highlights aspects of cellularization, activated transport mechanisms, interacting signaling pathways and extracted key genes impacting these processes.

### 3.1. Timing of Cellularization and Differentiation in Endosperm Subdomains Varies

Pre-/cellular morphology and gene expression patterns related to cell cycle regulation, cytoskeleton assembly and cell wall formation clearly showed a developmental gradient between the ETCs and Sync. Cellularization is more progressed in the ETCs, particularly in the 1L-layer and to a lesser degree in 2L, which is reflected by the expression of cell wall genes and is also consistent with previous studies on barley ETC development [30]. The preferential expression of alpha- and beta-expansins (α-/ß-EXP) point to activated cell elongation in the ETCs which correlates to the different cell shape in the 2L cell files that tend to expand in longitudinal direction. The 2L cells are not fully differentiated and a third layer of ETCs will be formed after the following rounds of anticlinal and periclinal cell divisions around 5 DAF, revealing that the cellular differentiation continues and a developmental gradient also exists within the ETC region. Remarkably, among the cell-wall-related genes expressed in ETCs, *HvEXPB4* and *HvKATAMARI1/MUR3,* two pectinesterases (*HvPEs*) and two pectin methylesterase inhibitors (*HvPMEIs*), are predicted to be highly ranked components of a gene regulatory network (GRN) controlled by *HvHK1* that coordinates regular ETC specification/differentiation [17]. They are highly connected with TCS elements (type-B RRs) and *HvSERK1* in the GRN, genes that are also enriched in ETCs in our dataset, and thereby support a link between phosphorylation modules and specific steps of primary wall formation of ETCs. Cell wall composition differs between ETCs and AL cells in cereal endosperms as reported by immunolocalization and Raman spectroscopy experiments in wheat grains [75]. Other than callose, 1,3-1,4-ß-glucans/hemicelluloses and arabinoxylans, heteroxylans are the dominating cell wall polysaccharides with different ratios between ETCs and AL cells, changing dynamically until late grain development. Genes involved in pectin esterification and metabolism stand out as enriched in ETCs at 3/4 DAF, which is somehow corroborated by immunolocalization experiments in barley grains using an LM19 antibody showing an accumulation of pectins in the ETC region, whereas no signals were detected in AL layers [76]. Transcriptome analysis of the manually dissected barley endosperm from the syncytial to the fully cellularized stage (3-8 DAF) unveiled multiple gene clusters associated with key stages of endosperm differentiation [30]. Further, co-expression networks of cell-wall-related genes and differentially expressed TFs identified potential regulators involved in the control of cell wall metabolism that cluster into two main groups with elevated expressions at early (3 to 4 DAF) and late (5-8 DAF) cellularization stages. Modules showing peaking expression at 3-4 DAF contain CES-like A/-E homologs, callose synthase, glycosyl transferases, hydrolytic and polysaccharide modifying enzymes, such as pectinesterases (PE/PEA/PME) and ß-glucanase, and several EXPs. Thereby coinciding with observations that callose and 1,3-1,4-ß-glucans are (transiently) present in initial syncytial walls at the onset of endosperm cellularization [77]. Our LCM-based analysis elucidated no significant differences between hemicellulose and callose synthesis in the subdomains, indicating that the deposition of both cell wall components was initiated in the ETC and Sync walls. Some specific genes attributed to ETC wall metabolism were also found in the early peaking modules of the complete syncytium, namely four *HvPE/PAEs* (HORVU3Hr1G091360, HORVU6Hr1G082990, HORVU5Hr1G114530, HORVU2Hr1G090330), *xyloglucan endotransglucosylase 10* and *endo-1,4-beta-xylanase* (HORVU4Hr1G028720, HORVU4Hr1G063790) and one gene encoding *EXPB2* (HORVU1Hr1G054240), reinforcing that modulation of the pectin matrix and cell expansion by cell-wall-loosening enzymes is essential for the biogenesis of ETCs. Modules of co-expression networks at early stages of syncytial cellularization contained a cluster of MADS and three HD-ZIP TFs from which four MADS genes (HORVU1Hr1G023620, HORVU1Hr1G029220, HORVU3Hr1G026650, HORVU5Hr1G095710) and one HD-ZIP (HORVU6Hr1G072810) were found to be preferentially expressed in Sync. They are tightly linked to Sync-specific *xyloglucan endotransglucosylase 5* and *glycosyl hydrolase 10* (HORVU4Hr1G028720, HORVU4Hr1G063790) associated with hemicellulose modification and turnover, but an exact role for the TFs in controlling cell wall formation in Sync alveoli would have to be proven experimentally. These examples for the separation of gene expression profiles in the syncytial subdomains underline the value of the dataset providing spatially resolved information.

The peripheral layer in Sync is mainly in a precellular stage, consisting of alveoli where the nuclei are lined by anticlinal cell walls, but periclinal cell walls have often not been built (Figure 1h,i), thereby indicating the progression from the syncytial to the cytokinetic status. The concerted expression of A- and D-type cyclins (*HvCYCA1;1/-3;1, HvCYCD3;2/-4;1/-6;1*), four cyclin-dependent kinase isoforms (*HvCDKC;1*), cytoskeleton-binding proteins (actin-, microtubule-binding) and G-protein signaling indicate an active cell cycle. Cell cycle progression is tightly controlled by the periodic activity of various combinations of cyclins and interacting CDKs, revealing a high complexity in cell cycle regulatory mechanisms, particularly during acytokinetic mitosis in early endosperm development to produce a syncytium and endoreduplication resulting in polyploid endosperm cells [78]. The majority of D-type cyclins are reported to control the G1 to S progressions, whereas A-type cyclins are deemed to regulate intra-mitotic and G2 to M transitions that allow cells to proceed into the mitotic phase. In the developing maize endosperm, the expression and protein analysis of A-, B-and D-type cyclins as well as A- and B-type CDKs separate two main expression patterns, activated CYCA1;1/CYCB1;3 and CDKB1;1 during mitotic cell divisions and CYCDs and CDKA;1 with constantly high expressions until endoreduplication [79]. The preferential expression of A- and D-type cyclin isoforms in the barley syncytium seem to be associated with the onset of mitotic cell divisions in the peripheral Sync layer via complex formation with HvCDKCs. In Arabidopsis, CDKCs were shown to inhibit cellular differentiation and prolong growth durations by influencing CYCB/-D expression as exemplified by *AtCDKC1;2* during leaf development [80]. Notably, ZmCYCD5 is exclusively apparent in the AL layer at 7 and 13 DAF, which does not undergo endoreduplication and thereby can be regarded as a regulator of AL cell division and differentiation coinciding with expression of *HvCYCD* genes in Sync. The expression of core cell cycle genes (*CYCB1;1/-2;2, CYCD2;2*) is suppressed in *tgw6*-mutants of rice, affected in the *TGW6* gene encoding an IAA-glucose hydrolase, that showed a prolonged syncytial phase and delayed cellularization of the early endosperm, possibly mediated by changing auxin levels [81]. Together, cytokinesis seems to be suppressed in the Sync layer by maintaining mitosis in late phases and preventing cellular differentiation. A long temporal interval between the termination of mitosis and the initiation of anticlinal cell walls/alveoli formation has been observed in barley endosperms (up to 48 h) [82], whereas this hiatus is much shorter in rice and maize [83]. Alveoli formation starts at intersections of radial microtubule systems (RMS) by the insertion of the phragmoplast that guides cell plate assembly between neighbored nuclei. Upregulation of five microtubuli-associated proteins (*HvMAP65/-70s*) that bundle and stabilize microtubuli during cytokinesis and nine kinesin microtubule motor proteins involved in microtubule-based movement and phragmoplast assembly might be associated with dynamic spindle microtubule rearrangement. Reorganization of the RMS is associated with elongation of anticlinal walls from alveoli towards the CCV [6] before periclinal walls will be formed to generate a first peripheral cell layer (Figure 1i,k). G-protein signaling-mediated vesicle trafficking is essential for the insertion and fusion of cell plates during the formation of initial syncytial walls. Several candidate genes important for G-protein signaling pathways in Sync were identified by our study, namely *HvRAB/ROP-GTPases*, *HvRho/ROP-GAPs* and *HvARF/ROP-GEFs* that seem to be involved in the development of syncytial walls. Notably, ROP-GTPases mediate auxin signaling pathways and regulate the subcellular distribution of PIN2 [84].

### 3.2. Nutrient Transport in the Young Endosperm

Transcriptome data revealed that active assimilated transport facilitated by an energy-coupled transporter, particularly for hexose sugars, sucrose and amino acids, has been established in ETCs at initial cellularization stages. This is expected, as sugars are needed for cell proliferation and endosperm growth. Moreover, hexose sugars provide precursors for cell wall biosynthesis/modification, which is intensified until the ETCs are fully differentiated. Metabolite profiling of developing ETCs by GC-MS displayed a high abundance of hexoses, such as fructose, glucose and glucose-6-phosphate, between 5–7 DAF and amino acids glutamine and lysine at 5 DAF [30], which is in line with the here observed transcriptional profile in ETCs. Sugars also play a role as inductive signals for transfer cell specification and differentiation as shown in maize BETL cells, where mutations of *ZmINCW2* or *ZmSWEET4c* lead to defective BETL differentiation and the activity of the BETL regulator ZmMRP-1 is stimulated by hexoses [85]. *HvSWEET11* is an ortholog of *AtSWEET12* and *ZmSWEET11*, both of which are reported to be sucrose transporters. In Arabidopsis, *SWEET12* is localized in phloem parenchyma cells and facilitates sucrose uptake in the phloem, whereas ZmSWEET11 is required for the retrieval of apoplasmic sucrose in the BETL layer in an interplay with extracellular invertase and ZmSUT1 [86]. A similar role for amino acid export can be assumed for the *HvBAT1-like* AAP (HORVU3Hr1G053090), whose Arabidopsis ortholog was shown to be localized in vascular tissues and a function in phloem unloading for the export into sink tissues is proposed [87]. Different isoforms of the ABC/PGP auxin transporter and PIN family members are reciprocally regulated in both subdomains. *HvPIN3* orthologs are preferentially expressed in ETCs, contrary to the higher expression of *HvPIN1/-2* in Sync. All of these genes are similar to maize ZMPIN1a/-b/-2/-3b/-10b homologs, which showed different localization patterns in the maize endosperm tissues, BETL, AL and SE [88]. The expression of different isoforms of the auxin transport machinery hints at auxin accumulation in both syncytial domains but is facilitated by different family members, which represents a nice example for the subfunctionalization of gene isoforms. Whether PIN3 isoforms are involved in early endosperm periclinal cell divisions and PIN1/-2 mainly in anticlinal ones remains a speculation and would be interesting to analyze with functional studies.

### 3.3. Signaling Pathways and Regulatory Modules Involved in Cell Fate Decisions

Hormone biosynthesis, metabolism and signaling differs significantly between syncytial compartments with ABA, ethylene and nodes of non-canonical auxin signaling dominating in ETC specification, whereas canonical auxin, GA and CK signaling is mainly activated in peripheral Sync layers. Identified key genes of hormonal pathways, peptide-receptor signaling and diverse TF families seem to interact, and mark transcriptional domains that direct the acquisition of cell identity. Preferential expression of putative ethylene receptors, a suite of ERF/AP2 TFs and biosynthesis genes (HvACCox1) in ETCs, imply that ethylene is an important signal for transfer cell (TC) specification. The complex formation of ethylene receptor HvETR2 with HvHP2, and HvHP2 with HvRR15 (Figure 6i), indicated that co-expressed TCS elements participate in common phosphorylation pathways that mediate ethylene signaling in ETCs. This is in line with a proposed role for ethylene in TC induction and development in *Vicia faba* cotyledons that has been uncovered by in vitro culture experiments [89] and the observation that the ethylene precursor ACC increased the number of cells forming CWIs during TC differentiation in tomato roots [90]. Previous results from transcriptome profiling of a time series of developing barley ETCs [30] showed that ethylene biosynthesis and signaling genes are highly activated in ETCs at 5 and 7 DAF and peaking ACC concentrations in the endosperm between 2–6 DAF, corroborating a pivotal role for ethylene in ETC differentiation. Similarly, ABA signaling is strongly activated in ETCs as implied by the expression of the receptor *HvPYR1/RCAR, HvPLDa1, HvPP2C* family genes, and DREB, MYB and WRKY TFs. In rice, ABA application to young grains enhanced cell division in the endosperm and increased the grain filling rate [91]. Significant concentrations of ABA are present in the endospermal pedicel/chalazal zone of maize kernels, which represents the maternal–filial boundary in kernels [92]. This is consistent with the analysis of the barley *seg8*-mutant which showed aberrant tissue differentiation in the central endosperm, particularly in the ETC region, a defect that was associated with changed ABA levels and signaling in filial grain tissues [93]. Other than ethylene and ABA, non-canonical auxin signaling elements are enriched in ETCs, i.e., auxin response genes, *HvAGO1/-4, HvAIR3* and *HvTPD1L* homologs, that were shown to be exclusively expressed in barley ETCs at different developmental stages in conjunction with potentially interacting TCS elements [17]. Data reveal that a specific branch of auxin regulation directs the cell division and differentiation of ETCs. Interestingly, most of the genes are highly ranked components of the GRN that specified direct targets of *HvHK1*. Compromised interactions of *HvAGO1, HvAIR3, HvPIN3* and peptide signaling (e.g., *HvTPD1Ls, HvSERK1*) in cells with perturbed ETC differentiation support the importance of these signaling nodes for the acquisition of ETC identity. Further, the nodes are proposed to be directly activated by type-B RRs, for which two *cis*-motifs have been identified as highly enriched in the top upregulated genes in ETCs (Table 1). Five *HvTPD1L* genes are outstandingly highly expressed in ETCs (TPM values 2000–10,000). TPD1/-L genes build a subgroup among cysteine-rich peptides (CRPs) and control megaspore mother cell proliferation in rice ovules and affect early endosperm development [94,95]. Arabidopsis TPD1 is processed into small, secreted CRPs and serves as a ligand for EMS1 and SERK1 LRR-RLKs [96]. Recently, ectopic expression of *TPD1* in Arabidopsis ovules induced the transcription of multiple auxin signaling/transport genes and affected core cell cycle genes (*CYCD3;3, CYCA2;3*) supporting a regulatory role in cell proliferation during early seed development [51]. A similar role for an RLK surface receptor and mobile CRP signaling in coordination of ETC cellularization can be anticipated. Prominent examples for an essential role of the CRP signaling in TC differentiation came from maize kernels, where the MEG1 peptide was verified as an essential regulator for BETL identity, and *ZmBETL1-4* genes were shown to be specifically expressed in the BETL layer [13,14]. In our analysis, MEG1-like or BETL-like genes were not found in the set of ETC-specific genes, probably because they are expressed at later stages of ETC differentiation, as maize BETL and MEG1 genes are reported to be expressed in BETLs between 8 and 20 DAF or in barley ETCs at 8 DAF (*HvBETL4*) [9,13,97]. The same applies for BETL-specific *ZmTCRR-1/-2* genes encoding atypical type-A RRs that lack the conserved aspartyl residue in the receiver domain and are also directly activated by ZmMRP-1 [10], although three type-A *HvRRs* are upregulated in ETCs during initial cellularization. High abundance of NAC and MYB *cis*-elements in the ETC-expressed genes overlaps with the expression of the TFs themselves. In Arabidopsis and Brachypodium, NAC and MYB family members were shown to control secondary wall biosynthesis [98,99], which is emblematic for ETC differentiation in forming cell wall ingrowths (CWIs) [37]. *ANAC48*, *ANAC14* and *MYB-related* genes were identified as putative transcriptional regulators of phloem-associated TCs in Arabidopsis leaves as *anac*- and *myb*-mutants showed reduced TCs in the veins of mature leaves [100]. Three orthologs of *ZmMRP-1* are upregulated in the ETC region at 3–4 DAF, which correlates to the increasing expression of *ZmMRP1/-like* genes from the coenocytic stage onwards until full differentiation of BETL cells. A LCM-based transcriptome analysis of distinct tissue types from 8 DAF maize kernels identified a BETL-specific regulatory module consisting of 93 target genes of ZmMRP1 [32]. Besides *MEG1*, *BETL* genes and *TCRR-1/-2*, a suite of genes involved in cell wall formation, lipid metabolism, hormone signaling and nitrate transport were uncovered as potential targets. Numerous barley genes, including several GDSL esterase/lipases, non-specific lipid transfer proteins (nsLTPs), among them three *HvEND1-like* (HORVU0Hr1G026630, HORVU1Hr1G012930, HORVU1Hr1G012940), *HvEXPB1*, *HvCESL*, *P-mannosidase*, nitrate transporter (*HvNRT1/PTRs*), *SNF1-related kinase*, *DREBB1*, *HvGH3-like* and *HvZF7* TF, are concomitantly upregulated in ETCs, implying the potential activation of similar target genes by HvMRP-1-like orthologs and functional conservation in barley ETCs, despite that co-expression networks have obviously been generated at a later stage of BETL/ETC differentiation.

Different hormone activities were elucidated in the transcriptome of the peripheral Sync layer, i.e., canonical auxin signaling, CK and GA metabolism/signaling. It is known that ratios between auxin, CK and GA levels and feedback interactions of them control cell division, cell elongation and the timing of cell differentiation in a fine-tuned balance. Auxin application to unfertilized ovules is able to initiate endosperm development in Arabidopsis and auxin is rapidly synthesized in the endosperm after fertilization, coinciding with a strong enhancement of auxin signaling until initial endosperm cellularization, as monitored by the auxin sensor R2D2 [101,102]. Excessive auxin biosynthesis and signaling prevents cellularization in defined regions of the young Arabidopsis endosperm [103]. Similarly in monocots, auxin strongly accumulates in maize kernels during early development [104] and the *defective kernel B18 (de18-mutant)*, affected in the biosynthesis enzyme ZmYUCCA1, displays strongly reduced IAA levels (<10%), which leads to defects in endoreduplication and smaller kernels [105]. The rice *tgw6*-mutant is a prominent example for auxin-mediated control of endosperm cellularization in monocots [81], despite that the reinvestigation of *TGW6* genes in rice and wheat indicates a role in floral organ/pollen ontogenesis rather than the regulation of free auxin levels in grains, where they are not expressed [106]. Auxin signaling antagonizes CK activities and it is documented that the ratio of IAA/CK is critical for tissue proliferation/differentiation and proper seed formation [107,108]. *ARF3* was shown to repress *IPT* and *LOG* family genes to diminish the CK activity during floral meristem determinacy in Arabidopsis [109]. In rice, the expression of *OsCKX4* is induced by exogenous auxin and is a direct target of *OsARF25* to promote CK degradation [110]. In Sync, auxin biosynthesis and signaling could play a similar role in antagonizing CK effects to prevent periclinal divisions in the syncytial layer before endosperm cellularization in centripetal direction starts. The enrichment of the ARF36 motif in promoters of the top DEGs in the Sync implies that numerous genes are targeted by ARF TFs, underlining the importance of ARF-mediated auxin signaling for controlling the timing of cellularization. Other Sync-specific TF genes belong to B3 domain proteins, bHLH and C2H2 protein families that are associated with auxin activities and control cell proliferation in reproductive organs of Arabidopsis. A critical role can be anticipated for *HvNKD1/2*, which maize orthologs are confirmed to regulate cell cycle and cell division by restricting auxin signaling in the AL layer and thus, to be a central regulator of AL specification/differentiation [24,59]. Further, application of the auxin transport inhibitor NPA perturbs the AL patterning in maize kernels, leading to the development of up to four AL cell layers instead of one [72]. Several studies have also established a link between LRR-RLKs and auxin signaling. *ERECTA (ER)* and their peptide ligands *EPIDERMAL PATTERNING FACTORS (EPF/EPFLs)* control cell division and expansion in an overlapping manner with auxins [111], and ER genes regulate the expression of *PIN1* in the shoot apical meristem (SAM) [112]. In our RNA-seq data, three *HvER1/-2/ER-LIKE1* and two *HvEPFL1/-2* orthologs were upregulated in Sync, supporting regulatory interactions that control cell divisions in the peripheral Sync layer. GA signaling acts downstream of auxin [113] and often synergistically in common pathways that promote cell elongation/expansion of early endosperm cells but also suppress tissue differentiation and developmental growth [114]. Three *HvGLABROUS* genes belonging to the type IV HD-Zip TF family were preferentially expressed in Sync. Type IV HD-Zip TFs were shown to dictate epidermal identity in the outermost peripheral layer of the rice syncytium, which is proposed to be a prerequisite for AL fate [35]. Several transcripts involved in GA synthesis, perception and signaling indicated pronounced GA influences in Sync. Besides SCL, GRAS and SPL TFs, five genes encoding barley GRFs (*HvGRFs*), and interacting factor *HvGIF3* were highly upregulated in Sync, representing orthologs of *AtGRF1 + 3* and *AtGIF1* and rice *OsGIF1*. GRFs are plant-specific TFs that control cell expansion in various organs and play a role in central developmental processes, including the formation of reproductive organs and seed development [63]. GRFs build a regulatory complex with GIF1, also named ANGUSTIFOLIA3 (AN3), and target *KNOX, SCL, MADS, HB* and *ARF* TFs from different dicot and monocot species, including barley *KNOX3*. Intriguingly, an important role for the initiation and maintenance of nematode-induced syncytium formation in root cells has been unraveled for *AtGRF1* and *AtGRF3* [115]. In contrast to the syncytial endosperm, pathogen-induced syncytium formation is a redifferentiation process, but confirms that GRFs are required for the reprogramming of cell identities and nuclear proliferation. TCP4 regulates the expression GRF/GIF and acts as a negative regulator of leaf cell proliferation [116]. TCP family TFs are specifically expressed in Sync, comprising *HvTCP4, HVTCP8* and *HvTCP15*, whose orthologs in Arabidopsis were shown to control endoreduplication by affecting genes involved in cell cycle regulation and to modulate auxin and GA responses [117,118,119].

Together, the concerted action of TF genes in the Sync revealed combinatorial molecular circuitries that promotes auxin and GA signaling, simultaneously diminishing CK activity. Regulatory loops directly activate auxin and GA biosynthesis genes, whereas CK biosynthesis is repressed and/or CK degradation is stimulated, probably to maintain hormonal balances until cellularization starts. These antagonistic auxin/GA-CK interactions might restrict cell proliferation and differentiation and retain the syncytial phase. This is an explanation for the developmental delay of Sync that will assume AL identity around 6/7 DAF compared to ETCs. There is a functional relationship between the development of ETCs and the other endosperm tissues, making it plausible that the ETC region has to be at least (partially) differentiated to ensure assimilation and signal transfer into the endosperm before AL and later, SE cell specification occurs. This is substantiated by the growth parameters of barley endosperm tissues determined by 3D imaging, showing that the functionality of ETCs has to be established before the endosperm growth with linear volume increasement was assessed [30]. Perturbed ETC/BETL development in cereal grains leads to the aberrant patterning of adjacent endosperm tissues, as exemplified for the maize *miniature (mn)*- and *sweet 4c*-mutants or for barley *HvHK1-RNAi* grains [17,120,121]. The repression of *HvHK1* impairs cell specification in the central ETC region, perturbs regular patterning of central endosperm cells and affects AL development. Only two instead of three layers are formed in transgenic grains, underlining that the growth coordination of tissue types is essential for regular endosperm development. 

Similar to auxin, the CK levels in cereal endosperms (rice, maize) increased after fertilization and showed the highest concentrations during initial stages of cellularization; in barley, a peak of iP and trans-zeatin concentrations was monitored at 4 DAF, i.e., at the progression from syncytial to cytokinetic phase, decreasing sharply thereafter [122,123,124,125]. The Sync subdomain consists of alveoli during the transition to cytokinesis. The process of insertion and fusion of periclinal wall fragments was partially initiated to complete cellularization (Figure 1h,i). The upregulation of key CK biosynthesis genes (*HvIPT3, HvLOG3*) and homeostatic enzymes points to activated CK signaling and balancing in Sync, coinciding with the peak in the CK levels at the onset of cellularization. An important role in induced CK signaling can be concluded for the putative CK receptor *HvHK5,* the only histidine kinase with a higher transcript abundance in Sync. Increased CK signaling could induce a shift in the hormonal balances towards CK vs. auxin/GA that triggers the start of cellularization in the syncytial region. Changes in the auxin/CK ratio were also reported to induce AL differentiation in maize grains [85]. A master regulator for the switch to cellular differentiation could be *HvKNOX11* (HORVU7Hr1G114650), which is the only class II HD KNOTTED TF distinctively expressed in Sync. The barley HVH21 motif bound by HvKNOX3 was the most enriched *cis*-element in the promoters of Sync-specific genes (Table 1), pointing to KNOX genes as key TFs in transcriptional cascades that target multiple genes during the onset of cytokinesis. HvKNOX3 controls spikelet development in barley and the *hooded* mutation induces the formation of extra flowers [126]. In several cases, KNOX genes were documented to repress GA biosynthesis and in turn, induce CK biosynthesis genes (IPTs), resulting in high CK/GA ratios that are required for meristem formation and organ differentiation in rice and maize shoots [127]. This prompted us to conclude that *HvKNOX11* might be the endosperm-specific isoform that causes a shift in hormonal balances, which promotes cellular differentiation in the peripheral Sync layer by releasing the ‘block’ of cellularization—maintained by auxin, GA and interacting TFs—to complement cytokinesis and initiate AL differentiation. A positive correlation between the duration of the syncytial phase and endosperm/seed size has been shown for rice in several cases [81] and might inhibit premature cellularization of the syncytium, in our case preventing cellularization before the functionality of ETCs in nutrient and signal transfer is established.

## 4. Materials and Methods

### 4.1. Plant Material

*Hordeum vulgare* cv. ‘Barke’ was grown in greenhouses at 18 °C with 16 h light and 60% air humidity. Flowers were tagged as described [47] and grains were harvested at 3–4 DAF. 

### 4.2. Light, Confocal and Transmission Electron Microscopy

Cross-sections (2 mm thick) from the central region of 3/4 DAF were used for microwave-assisted aldehyde fixation, substitution and embedding as described [30]. Semi- and ultra-microtomy, as well as histological and ultrastructural analyses, were performed as previously described. [128]. Cell wall staining was carried out with Calcoflour white (Sigma-Aldrich, Taufkirchen, Germany) 1 mg/mL in 50 mM phosphatebuffer, pH 7.2. Nuclei were stained with 1 mg/mL DAPI solution. Fluorescence was recorded in a LSM780 confocal laser scanning microscope (Carl Zeiss, Jena, Germany) using a 405 nm laser for excitation in combination with a 410–500 nm bandpass for detection.

### 4.3. Laser Capture Microdissection, Sample Preparation and RNA-Seq

Sample processing for RNA-seq was performed as described in detail by Brandt and colleagues [129]. Briefly, grains from Barke plants were harvested at 3/4 DAF, immediately frozen in liquid nitrogen, glued onto sample plates by O.C.T™ medium and serial cryosections (20 µm) were mounted on RNAse-free PEN membrane slides (MMI, Eching, Germany). PEN membrane slides were dried at −20 °C and the MMI Laser Cell Cut system was used to isolate the ETC region and the peripheral syncytium (Sync). Usually between 40–50 LCM-captured sections from five different grains were pooled for each biological replicate. RNA was isolated by using the Absolutely RNA Nanoprep Kit (Agilent, Waldbronn, Germany) and the mRNA was amplified by one round of in vitro transcription using the MessageAmp^TM^ aRNA kit (Invitrogen, Carlsbad, CA, USA). Antisense RNA was used for library preparation with Illumina TrueSeq RNA kit V2 (Illumina, San Diego, CA, USA). Libraries were sequenced on HiSeq 2500 (Illumina) to generate 100-base pair (bp) paired-end reads. On average, more than 43 million reads with a mean Phred-quality score (PF) of 37.5 were generated per sample.

### 4.4. Read Mapping and Transcriptome Analysis

RNA-seq reads were processed using CASSAVA v1.8 and trimmed as recommended in the manufacturer’s instructions. Cleaned reads were mapped against the barley genome [130] using HISAT2 and the raw read counts were normalized to transcript per million (TPM) expression levels (Table S1). DESeq2 [131] was used for the identification of differentially expressed genes (DEGs) according to a log2-fold change > 1 and false discovery rate (FDR) < 0.05 (Supplementary Table S2). Barley sequence information is publicly available in the Barlex genome explorer (https://apex.ipk-gatersleben.de/apex/f?p=284:10, accessed on 2 July 2022). The analysis focused on high confidence (HC) genes, and low confidence (LC) genes were excluded to avoid confounding effects from pseudogenes and low-quality gene models. Analysis of the GO term enrichment in DEGs was performed by agriGO (http://bioinfo.cau.edu.cn/agriGO/analysis.php, accessed on 2 July 2022) using the SEA tool applying a hypergeometric test with FDR correction (adjusted *p* value < 0.05). Heatmaps for gene expression data were generated using the online software TBtools [132].

The RNA-seq data of this study were deposited in the Sequence Read Archive (SRA) database under the accession number PRJNA926383 (Bioproject ID).

### 4.5. Analysis of Cis-Motif Enrichment

To identify enriched *cis*-elements in genes highly expressed in either ETC or Sync (top500 DEGs), we employed the motif finding algorithm HOMER [67]. The HOMER algorithm was run with the following parameters: motif lengths 8, 9, 10, 11, 12, 13, 14, 15, number of motifs 200, mismatches 2, and max normalization iterations 160. Identified motifs were checked against motif databases and either scored as known or unknown motifs.

### 4.6. In Situ Hybridization

Barley grains were fixed in 4% (*v*/*v*) paraformaldehyde in phosphate-buffered saline, pH 7.3, overnight at 4 °C. After dehydration by an ethanol series, samples were passed through a graded ethanol–methacrylate series and polymerized for at least 48 h in UV light (20 °C). Cross-sections (7 mm) were prepared and mounted on silane-coated slides (Sigma-Aldrich). Gene-specific fragments were amplified by PCR using gene-specific primers containing T3- and T7-promoter sequences (Table S6). Fragments were labelled with Digoxigenin (DIG) by in vitro transcription using T3- and T7-polymerase according to the manufacturer’s instructions (Roche Diagnostics, Mannheim, Germany). After the purification of riboprobes, the efficiency of DIG labelling was verified by dot blotting and the probes (100 ng RNA) were denatured and treated with RNAse inhibitor for hybridization

Hybridization and immunological detection were performed after Drea and colleagues [133]. The hybridization signals were detected by an alkaline phosphatase-conjugated DIG antibody and visualized with 4-nitroblue tetrazolium chloride (NBT) and 5-bromo-4-chloro-3-indolyl phosphate (BCIP, Roche, Penzberg, Germany).

### 4.7. Bimolecular Fluorescence Complementation

Sequences of barley TCS elements (REC domain of HvHK1, HvHP2, mutated HvHP2, HvRR1, HvETR2, HvHP1, HvRR15) were codon optimized for expression in *Nicotiana benthamiana* and cloned into SPYNE-35S and SPYCE-35S vectors [134]. The mutated ∆HvHP2 element was generated by replacing the histidyl phosphoacceptor site (H76) with leucine (L76). Vectors were introduced in the Agrobacterium tumefaciens strain C58C1:pGV2260 by electroporation for the infiltration of tobacco leaves of 5–6-week-old plants. The agroinfiltration of constructs was supplemented with the silencing suppressor HC-Pro and acetosyringone (1:1000) and the expression of the fusion proteins was examined with Carl Zeiss CLSM 780 after 2 days of incubation. YFP was excited with a 514 nm argon laser and the specificity of the signals was confirmed by a lambda signature and the expression of TCS elements in tobacco leaves was verified by Western blots (Figure S2) as described by Gahrtz & Conrad [135].

## 5. Conclusions

Together, this tailored approach to capture the transcriptomes of syncytial subdomains of the endosperm —just at the onset of cellularization—provides new insights into spatial molecular mechanisms directing ETC and AL fate acquisition. The data reveal regulatory pathways that control the duration of the syncytial stage and initial cell differentiation, which are important phases in endosperm development as the cell number, endosperm size and finally, grain yield is largely determined during this period. The ETC identity is associated with distinct regulatory nodes, such as TCS-mediated phosphorylation pathways, peptide and hormone signaling (ethylene, ABA and non-canonical auxin). In the peripheral syncytial layer, the fine-balancing between repressory modules that prevent cellularization—with auxin and GA and interacting TFs as main triggers—and differentiation-promoting modules—with CK signaling and potential key TFs—governs AL cell specification. Key candidate genes controlling cell cycle regulation, timing of cytokinesis and cellular differentiation were identified, but the dataset can similarly be queried for any other pathway and/or candidate genes of interest. The analogy of developmental programs and key determinants of endosperm tissue differentiation in other cereal crops, such as rice, maize and wheat, supports the conservation of molecular mechanisms controlling seed development in monocots. Subsequently, our dataset provides an essential framework for the initial endosperm differentiation in barley, but may be indispensable for comparative studies with other grain crop cereals.

## Figures and Tables

**Table 1:** Top12 enriched *cis*-regulatory motifs in ETCs or Sync. Promoters of the top 500 DEGs were evaluated by HOMER. Motifs are ranked by enrichment factors.

			*ETC*		*Sync*	
*Motif Logo*	*Name*	*TF Family*	*log P-val*	*Enrich. Factor*	*log P-val*	*Enrich. Factor*
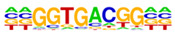	HVH21 (HD-KNOTTED)/Hordeum vulgare	HD-Knotted	-	-	−3.55 × 10^4^	13.28
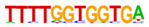	ERF015/MA1265.2/Jaspar	AP2ERF	−4.38 × 10^4^	10.2	-	-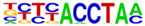	MYB41/col-MYB41-DAP-Seq/Homer	MYB	−3.36 × 10^4^	8.71	-	-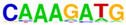	SEP3/Arabidopsis-Flower-Sep3-ChIP-Seq/Homer	MADS	−8.04 × 10^3^	1.09	−4.92 × 10^4^	8.23
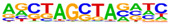	MYB3/MA1038.1/Jaspar	MYB	−3.07 × 10^4^	8.16	-	-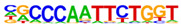	TAGL1/Tomato-TAGL1-ChIP-Seq/Homer	MADS	-	-	−3.22 × 10^4^	7.69
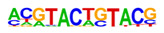	SPL13/MA1321.1/Jaspar	SPL	-	-	−3.82 × 10^4^	7.29
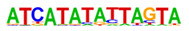	CRC/col-CRC-DAP-Seq(GSE60143)/Homer	C2C2 YABBY	−5.78 × 10^4^	7.17	-	-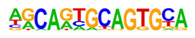	At5g04390/col200-At5g04390-DAPseq/Homer	C2H2	-	-	−4.73 × 10^4^	6.68
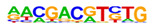	HY5/colamp-HY5-DAP-Seq/Homer	bZIP	−8.89 × 10^3^	1.12	−3.35 × 10^4^	6.41
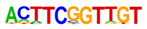	MYB101/MA1173.1/Jaspar	MYB	−2.80 × 10^4^	6.34	-	-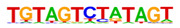	AT1G04880/colamp-DAP-Seq/Homer	ARID	-	-	−3.32 × 10^4^	6.34
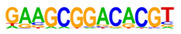	ARF36/MA1695.1/Jaspar	ARF	-	-	−4.39 × 10^4^	6.34
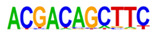	bZIP910/Antirrhinum majus/AthaMap	bZIP	-	-	−2.98 × 10^4^	6.28
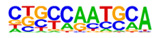	NFYB/MA0502.2/Jaspar	NF	−3.37 × 10^4^	6.16	-	-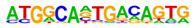	STZ/MA1372.1/Jaspar	C2H2	-	-	−2.93 × 10^4^	6.14
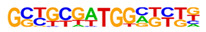	HAP3/col-HAP3-DAP-Seq/Homer	CCAATH AP3	-	-	−4.26 × 10^4^	5.92
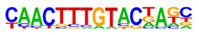	At4g38000/col-At4g38000-DAP-Seq/Homer	C2C2 Dof	-	-	−3.59 × 10^4^	5.58
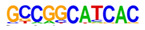	ERF6/MA1006.1/Jaspar	AP2ERF	−2.78 × 10^4^	5.50	-	-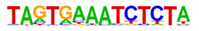	CCA1/MA0972.1/Jaspar	MYB related	−4.97 × 10^4^	5.37	-	-
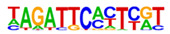	AT5G45580/colamp-DAP-Seq/Homer	G2 like	−4.76 × 10^4^	5.12	-	-
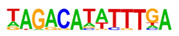	LCL1/MA1187.1/Jaspar	MYB related	−4.56 × 10^4^	4.97	-	-
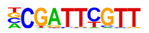	ARR1/MA0945.1/Jaspar	RR type B	−2.85 × 10^4^	4.93	-	-
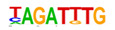	ARR10/MA0121.1/Jaspar	RR type B	−4.04 × 10^4^	4.90	-	-

## Data Availability

Raw data of this study have been deposited in Sequence Read Archive (SRA) database under the accession number PRJNA926383 (Bioproject ID).

## Supplementary Materials

The following supporting information can be downloaded at: https://www.mdpi.com/article/10.3390/plants12081594/s1, Table S1. TPM values of barley HC and LC genes in all samples with barley annotation and annotation of closest Arabidopsis orthologs. Table S2. DEGs between ETCs and Sync at 3/4 DAF (HC + LC; HC genes). Table S3. GO term-enrichment in DEGs between ETCs and Sync at 3/4 DAF (FDR < 0.05). Table S4. DEGs in functional categories. Table S5. *Cis*-motifs in promoter regions of DEGs (Top 500 genes). Table S6. Gene-specific primer sets for DIG-labelling of T3 and T7 probes used for in situ hybridization. Figure S1. Heat map display of differentially expressed TCS genes between ETCs and Sync. Figure S2. Expression confirmation of TCS elements in tobacco leaves for BiFC assays by Western Blot and lambda signatures confirming specificity of YFP signals.
