# Supplementary material for: Dissection of Developmental Programs and Regulatory Modules Directing Endosperm Transfer Cell and Aleurone Identity in the Syncytial Endosperm of Barley
Source: Plants (Basel). 2023 Apr 10;12(8):1594. doi: 10.3390/plants12081594 (PMC10142620; doi:10.3390/plants12081594)
Supplement: Supplementary file 1 [file plants-12-01594-s001.zip › plants-2254683-Figures S1 and S2.pdf]

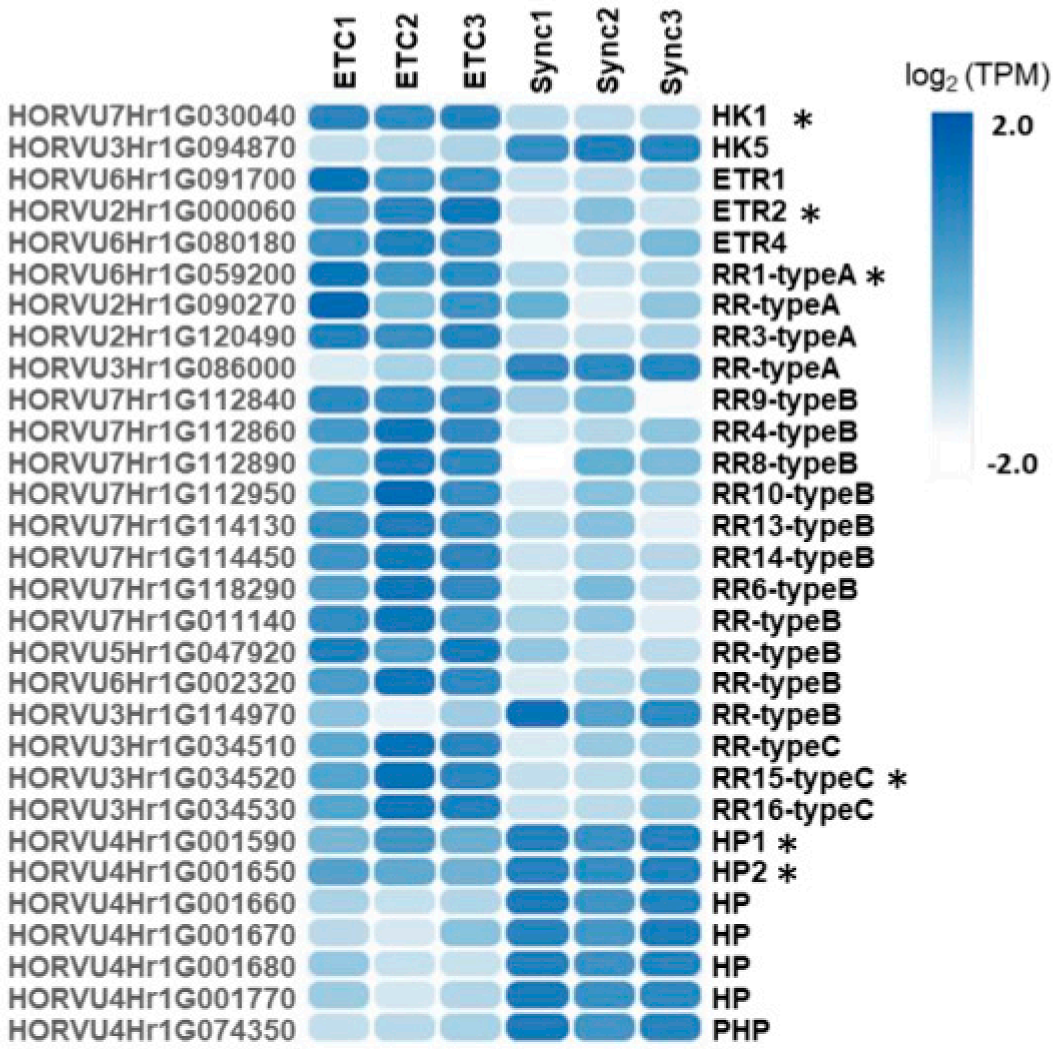

Figure S1. Heat map display of differentially expressed TCS genes in ETCs and Sync. Log$_2$-transformed TPM values are given for biological replicates, color code is row-scaled. Stars mark genes used in BiFC assays.

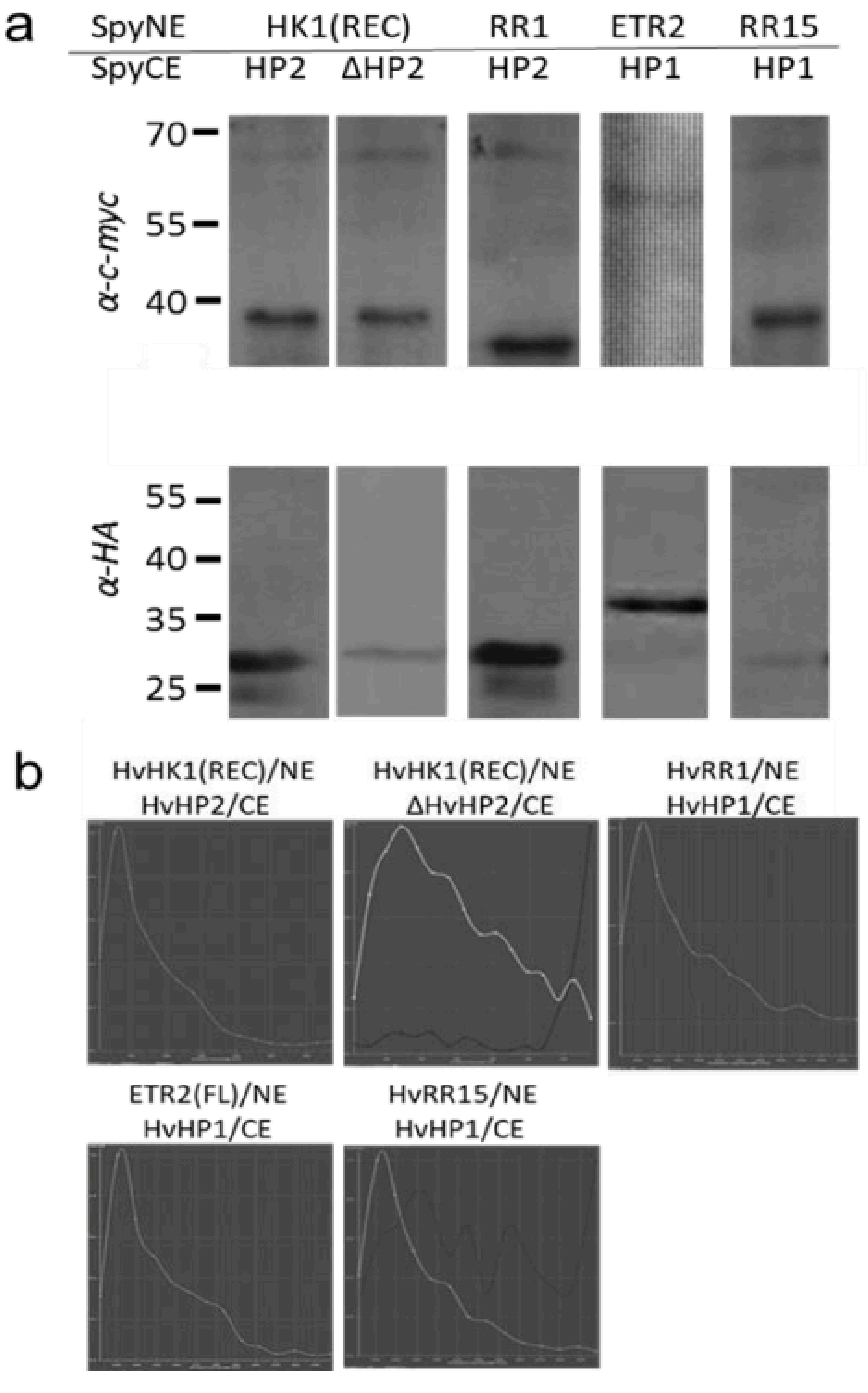

Figure S2. Protein expression and Lambda screening of YFP signals. a) Detection of expressed TCS elements in tobacco leaves by Western blot with specific antibodies, α-c-myc for SpyNE-elements, α-HA for SpyCE-elements; b) Lambda screening with YFP peaks at 527 nm.
